# New insights into the prevalence of depressive symptoms and depression in rheumatoid arthritis – Implications from the prospective multicenter VADERA II study

**DOI:** 10.1371/journal.pone.0217412

**Published:** 2019-05-28

**Authors:** Matthias Englbrecht, Rieke Alten, Martin Aringer, Christoph G. Baerwald, Harald Burkhardt, Nancy Eby, Jan-Paul Flacke, Gerhard Fliedner, Ulf Henkemeier, Michael W. Hofmann, Stefan Kleinert, Christian Kneitz, Klaus Krüger, Christoph Pohl, Georg Schett, Marc Schmalzing, Anne-Kathrin Tausche, Hans-Peter Tony, Jörg Wendler

**Affiliations:** 1 Freelance Healthcare Data Scientist, Eckental, Germany; 2 Department of Internal Medicine 3 –Rheumatology and Immunology, Universitätsklinikum Erlangen, Friedrich-Alexander-Universität Erlangen-Nürnberg (FAU), Erlangen, Germany; 3 Internal Medicine, Rheumatology, Schlosspark-Klinik, Charité, University Medicine Berlin, Berlin, Germany; 4 Medicine III, University Medical Centre Carl Gustav Carus at the TU Dresden, Dresden, Germany; 5 Department of Medicine, Neurology and Dermatology, Rheumatology Unit, University Hospital Leipzig, Leipzig, Germany; 6 Division of Rheumatology, University Hospital Frankfurt am Main Goethe-University, Frankfurt/Main, Germany; 7 Biostatistics, AMS Advanced Medical Services GmbH, Mannheim, Germany; 8 Medical Department—Rheumatology, Roche Pharma AG, Grenzach-Wyhlen, Germany; 9 Rheumatology, Rheumatologische Schwerpunktpraxis, Osnabrueck, Germany; 10 Medical Department—Rheumatology, Chugai Pharma Europe Ltd., Frankfurt/Main, Germany; 11 Rheumatologische Schwerpunktpraxis, Drs. Kleinert, Rapp, Ronneberger, Schuch u. Wendler, Rheumatology, Erlangen, Germany; 12 Rheumatology/ Clinical Immunology Dept, Internal Medicine II, University Hospital Wuerzburg, Germany; 13 Rheumatologische Schwerpunktpraxis, Schwerin, Germany; 14 Medicine/Rheumatology/Immunology, Klinikum Südstadt Rostock, Rostock, Germany; 15 Rheumatology, Praxiszentrum St. Bonifatius, Muenchen, Germany; University of Twente, NETHERLANDS

## Abstract

**Objectives:**

To investigate the prevalence of depressive symptoms in rheumatoid arthritis (RA) patients using two previously validated questionnaires in a large patient sample, and to evaluate depressive symptoms in the context of clinical characteristics (e.g. remission of disease) and patient-reported impact of disease.

**Methods:**

In this cross-sectional study, the previously validated Patient Health Questionnaire (PHQ-9) and Beck-Depression Inventory II (BDI-II) were used to assess the extent of depressive symptoms in RA patients. Demographic background, RA disease activity score (DAS28), RA impact of disease (RAID) score, comorbidities, anti-rheumatic therapy and antidepressive treatment, were recorded. Cut-off values for depressive symptomatology were PHQ-9 ≥5 or BDI-II ≥14 for mild depressive symptoms or worse and PHQ-9 ≥ 10 or BDI-II ≥ 20 for moderate depressive symptoms or worse. Prevalence of depressive symptomatology was derived by frequency analysis while factors independently associated with depressive symptomatology were investigated by using multiple logistic regression analyses. Ethics committee approval was obtained, and all patients provided written informed consent before participation.

**Results:**

In 1004 RA-patients (75.1% female, mean±SD age: 61.0±12.9 years, mean disease duration: 12.2±9.9 years, DAS28 (ESR): 2.5±1.2), the prevalence of depressive symptoms was 55.4% (mild or worse) and 22.8% (moderate or worse). Characteristics independently associated with depressive symptomatology were: age <60 years (OR = 1.78), RAID score >2 (OR = 10.54) and presence of chronic pain (OR = 3.25). Of patients classified as having depressive symptoms, only 11.7% were receiving anti-depressive therapy.

**Conclusions:**

Mild and moderate depressive symptoms were common in RA patients according to validated tools. In routine clinical practice, screening for depression with corresponding follow-up procedures is as relevant as incorporating these results with patient-reported outcomes (e.g. symptom state), because the mere assessment of clinical disease activity does not sufficiently reflect the prevalence of depressive symptoms.

**Clinical trial registration number:**

This study is registered in the Deutsches Register Klinischer Studien (DRKS00003231) and ClinicalTrials.gov (NCT02485483).

## Introduction

Rheumatoid arthritis (RA) is a chronic inflammatory disease of the musculoskeletal system that leads to pain, swelling and progressive joint destruction via increased activity of pro-inflammatory cytokines [1]. Although a variety of conventional and biological disease modifying anti-rheumatic drugs are available to control disease activity in RA, the disease is still challenging and usually requires life-long treatment. Furthermore, it is associated with a substantial reduction of overall physical and emotional well-being not only due to articular symptoms including pain and loss of physical function but also as a result of fatigue and impaired quality of sleep [1, 2]. This disease burden can affect the patient’s quality of life and socioeconomic outcomes including workplace absenteeism and presenteeism (low workplace productivity) [3–5]. For patients with RA, a greater loss of workplace productivity has been associated not only with functional disability but also with depressive symptoms [6]. Moreover, depression has also been associated with high costs of absenteeism and presenteeism in a wide range of countries across a variety of socioeconomic statuses [7]. Against this background, the challenge of depression as a comorbidity of RA becomes evident: in combination, these disorders pose an even more considerable burden for patients and are likely to result in more frequent and longer periods of absence. In clinical practice, depressive symptoms and affective disorders are common in RA [8–10] with a recent meta-analysis reporting 16.8% of RA patients as having a major depressive disorder [11]. Furthermore, Dougados et al. found depression to be the most frequent comorbidity in RA (15%) [12]. Thus, detecting and addressing depression in patients with RA needs to be a part of patient care. This means, applying standardized procedures to screen for depression and in the case of positive screening results, a follow-up with a full diagnostic assessment [13].

A recent meta-analysis emphasized the need for screening tools to be validated against a clinical interview as gold standard in order to assess depressive symptoms [11]. Following these recommendations, we previously evaluated the psychometric properties of the Patient Health Questionnaire 9 (PHQ-9) [14], the Beck Depression Inventory II (BDI-II) [15] and the World Health Organization 5-Item Well-Being Index (WHO-5) [16] against a structured patient interview using the Montgomery-Ǻsberg Depression Rating Scale (MADRS) in RA patients [17]. Within this validation study (VADERA I), we were able to show that the PHQ-9 and BDI-II are valid and reliable for depression screening in patients with RA [17]. The aim of the present study–VADERA II–was to use the PHQ-9 and BDI-II to determine the prevalence of depressive symptoms in RA patients in clinical practice and to subsequently evaluate the role of covariates such as disease impact measured using the Rheumatoid Arthritis Impact of Disease (RAID) questionnaire. Although a number of instruments have been introduced to assess depressive symptoms in rheumatology [11, 18], to the best of our knowledge, VADERA II is the first study within its field to have explicit validation of self-reporting tools prior to determining prevalence of depressive symptoms.

## Methods

### Description of the patient sample

VADERA II was a cross-sectional study. Patients were enrolled from October 2014 to September 2015 at centres in Germany. Inclusion criteria included a documented diagnosis of RA according to the ACR/EULAR 2010 criteria, age of ≥18 years, the ability to complete the questionnaires and scheduling of a RA consultation at one of the participating sites. To avoid sampling bias and to assure a representative study population, investigators were informed of the importance of enrolling a practice-oriented and representative sample of RA patients. There were no exclusions of patients due to RA disease activity, medical treatment or comorbidities. All sites were requested to document the reasons for non-participation of patients. Institutional review board approval was obtained from the ethics committee of the Medical Faculty of the Friedrich-Alexander University Erlangen-Nuremberg (4431), and all patients completed an informed consent form before participation. This study is registered in the Deutsches Register Klinischer Studien (DRKS00003231) and ClinicalTrials.gov (NCT02485483).

The study centres were all members of the working group for the evaluation of systemic effects of RA (SYRA). The VADERA II study was conducted in 10 centres representing a spectrum of clinical settings including large academic medical centres, community hospitals and private rheumatology practices across Germany. Study participation consisted of a single clinic visit, which included self-completion of the depression questionnaires.

Sample size considerations for this study were based on a review of the medical literature for previously reported depression prevalence rates in RA patients. The reported prevalence of depression in RA patients ranged from 6.4% as reported by Covic et al. in 2012 [19] to 41.5% as reported by Isik et al. in 2007 [20]. Thus, it was assumed that with the enrolment of 1000 patients, between 64 and 415 patients in the study would have depressive symptomatology, which would be adequate for the planned analyses.

### Study-related procedures and questionnaire cut-points for depressive symptoms

Data collection in VADERA II was cross-sectional. All data collected in connection with the VADERA II study were entered in an electronic case report form (eCRF) including the paper-based BDI-II and PHQ-9 questionnaires [21] and the RAID questionnaire (consisting of seven items: pain, functional disability, fatigue, sleep, physical well-being, emotional well-being, coping) [2, 22], all of which were completed during the patient’s study visit. In addition, demographic data (age, gender, height, weight, family status, educational status and employment status), RA disease activity according to the Disease Activity Score based on 28 joints and erythrocyte sedimentation rate (DAS28 (ESR)), laboratory and serologic parameters (CRP, haemoglobin, ESR, rheumatoid factor and anti-citrullinated protein antibody (ACPA)), and comorbidities were recorded. Furthermore, information on treatment with anti-rheumatic therapy in categories (conventional and biological Disease-Modifying Anti-rheumatic Drugs (cDMARDs/bDMARDs), glucocorticoids, nonsteroidal anti-inflammatory drugs (NSAIDs), opioids, other medication groups) were recorded as well as anti-depressive treatment in categories (tricyclic antidepressants (TCAs), serotonin or norepinephrine reuptake inhibitors (SNRIs), monoamine oxidase inhibitors (MAOIs), psychotherapy, other). Comorbidities at the time of study participation and potential adverse events were also documented. Standardized cut-offs of the PHQ-9 and the BDI-II as defined by the questionnaire developers [15, 23] were applied.

Cut-off values for positive depressive symptomatology were PHQ-9 ≥5 or BDI-II ≥14 which corresponds to the presence of at least mild depressive symptoms. Numbers for the frequency of depressive symptomatology were derived by frequency analysis. Point prevalence rates (patients with positive depressive symptoms / total study population) and their 95% confidence intervals (CI) were calculated using the Clopper-Pearson (exact) method.

### Covariates of depressive symptoms

The risk of depressive symptomatology was further evaluated by taking into account the effects of other covariates, which were included in univariate and multiple logistic regression analysis. The potential effects of covariates on the risk of depressive symptomatology were evaluated by the odds ratio (OR) and 95% CIs. All potentially important covariates were included in a full model, and a step-down procedure was used to evaluate the impact of the covariates in the model. The corresponding variables (and arbitrary cut-offs) of interest were as follows: Age (<60 years, ≥60 years), sex (0 = female, 1 = male), family status (0 = single, 1 = family), BMI kg/m^2^ (0 = <25, 1 = ≥25), education (ten years of school education or more = 1, less than ten years of school education = 0), employment (other = 0, 1 = employed), duration of RA (continuous variable), DAS28 (ESR) remission (<2.6, ≥2.6), RAID patient acceptable symptom state (PASS) (i.e. RAID ≤2 vs. RAID >2) [24], presence of chronic pain (0 = no, 1 = yes), presence of a comorbidity other than mental illness or chronic pain (0 = no, 1 = yes), and current medication–cDMARD (0 = no, 1 = yes), bDMARD (0 = no, 1 = yes), glucocorticoids (0 = no, 1 = yes), NSAIDs (0 = no, 1 = yes), opiates (0 = no, 1 = yes). Due to indications of multicollinearity in the multiple logistic regression when referring to RAID single numerical rating scales, we used the PASS criterion for the total score as the outcome of clinical relevance.

The potential effects of RAID single items on the risk of depressive symptomatology were measured by the OR and 95% CIs addressing distinct symptoms in the univariate analyses.

Sensitivity analyses were conducted using cut-off points for moderate depressive symptoms as previously defined by the developers of the PHQ-9 and BDI-II questionnaires. In order to evaluate the impact of the cut-off values of the PHQ-9 and the BDI-II on depression prevalence and the essential covariates, the cut-off values for moderate depressive symptomatology were set to a summary score ≥10 for the PHQ-9 questionnaire and a summary score of ≥20 for the BDI-II questionnaire, respectively.

All adverse events (AE), patients experienced between informed consent and completion of questionnaires were recorded, independent of AE severity or relationship to treatment. Continuous descriptive results are presented as mean (standard deviation; SD) if not stated otherwise. Descriptive analysis of nominal characteristics was conducted by referring to frequency analysis. Statistical analyses were computed using SAS Version 9.4 (SAS; Cary, NC, USA). The study results are reported according to the STROBE guidelines for observational studies [25].

## Results

### Patient sample

A total of 1015 patients with RA had given informed consent to the VADERA II study and 1004 patients (99%) completed at least one of the depression questionnaires and were included in the analysis. During this cross-sectional study, no serious AEs or adverse drug reactions occurred. Complete questionnaires (all questions answered) were available for evaluation of the PHQ-9 in 975 patients (97.1%) and the BDI II in 971 patients (96.7%). For the 36 of 1051 screened RA patients (3%) who did not complete the two depression questionnaires, the most common reasons were: ‘not enough time’ and ‘questions too personal’.

### Patient characteristics

Mean patient age was 61.0 (SD 12.9) years; 75.1% were female. The most common family status was living with a partner (58.4%), followed by single/living alone (23.5%), or living with partner and children (16.0%). Amongst others, 52.1% of the patients reported being retired, 39.4% were employed at the time of study participation.

Mean duration of RA in the study population was 12.2 (SD 9.9) years. Serology results indicated that 52.7% of the patients were rheumatoid factor (RF) positive and 61.2% ACPA positive.

**Fig 1:**
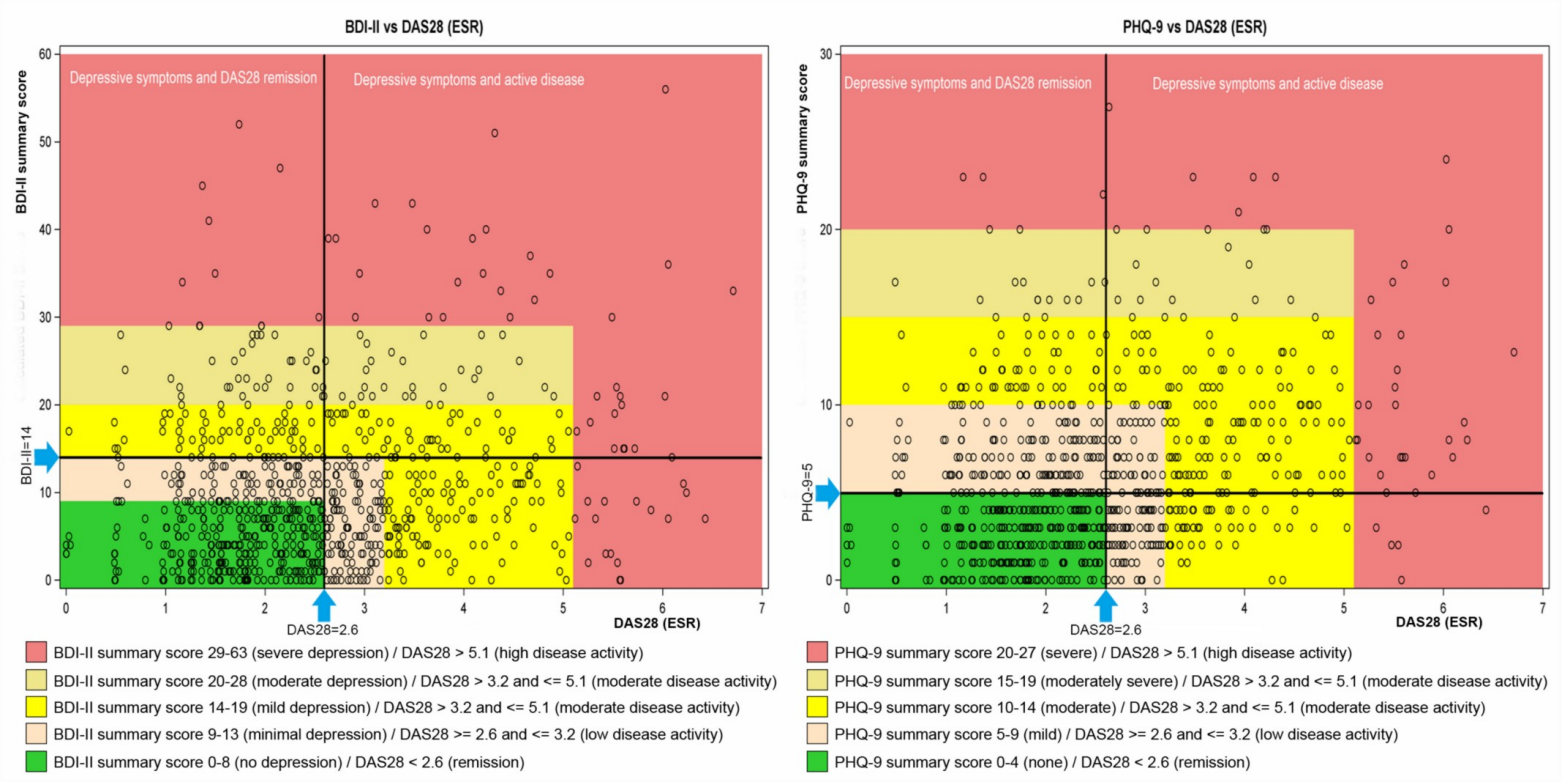
A/1B BDI-II and PHQ-9 scores by DAS28 (ESR). Legend: (Fig 1A) DAS28 (ESR) scores by BDI-II score with black lines showing cut-point for mild depression and low disease activity. (Fig 1B) DAS28 (ESR) scores by PHQ-9 score with black lines showing cut-point for mild depressive symptoms and low disease activity.

Mean DAS28 (ESR) was 2.5 (SD 1.2) while the median number of both swollen joints and tender joints was 0. The majority of patients (59.1%) were in DAS28 remission (DAS28 <2.6). ACR/EULAR Boolean remission [22] was reported for 25.6% of patients. DAS28 remission was found in 50.8% of patients having at least mild depressive symptoms in the PHQ-9 or the BDI-II (see Fig 1A and 1B). On average, the mean RAID score was 3.6 (SD 2.2) with 269 patients (27.0%) fulfilling RAID PASS with a total score ≤2 and 728 patients (73.0%) with a RAID score >2.

Presence of a comorbidity at time of participation was reported by 60.9% of patients (58.6% of patients excluding mental illness). The most common comorbidities were cardiovascular disease (42.7%) including hypertension (38.1%), diabetes mellitus (9.6%), chronic pain due to conditions other than RA (9.4%) and chronic respiratory diseases (8.0%). Of the total study population, 7.5% were receiving anti-depressive therapy.

Details on sample characteristics are shown in Table 1.

**Table 1:** Study population characteristics.

	PHQ-9 or BDI-II indicating depressive symptoms	PHQ-9 or BDI-II not indicating depressive symptoms	Total
Characteristics	(N = 556)	(N = 448)	(N = 1004)
Years of age [Mean (SD)]	60.2 (12.2)	62.0 (13.5)	61.0 (12.9)
Gender [n (%)]			
Female Male	429 (77.2%) 127 (22.8%)	325 (72.5%) 123 (27.5%)	754 (75.1%) 250 (24.9%)
Duration of RA in years [Mean (SD)]	12.3 (10.0)	12.1 (9.6)	12.2 (9.9)
Disease Activity Scores			
DAS28 (ESR) Score [Mean (SD)]	2.8 (1.3)	2.2 (1.0)	2.5 (1.2)
Boolean Remission [n (%)]	88 (16.3%)	162 (37.2%)	250 (25.6%)
RAID score [Mean (SD)]	4.6 (1.9)	2.3 (1.7)	3.6 (2.2)
Current RA therapy [n (%)]			
cDMARDs	441 (80.6%)	364 (82.2%)	805 (81.3%)
Glucocorticoids	303 (55.4%)	198 (44.7%)	501 (50.6%)
bDMARDs	269 (49.0%)	197 (44.5%)	466 (47.0%)
NSAIDs	233 (42.5%)	131 (29.6%)	364 (36.7%)
Opioids	36 (6.5%)	10 (2.3%)	46 (4.6%)
Presence of a comorbidity [n (%)]	360 (64.7%)	251 (56.0%)	611 (60.9%)
Cardiovascular diseases	239 (43.0%)	190 (42.4%)	429 (42.7%)
Diabetes mellitus	54 (9.7%)	42 (9.4%)	96 (9.6%)
Other chronic pain	73 (13.1%)	21 (4.7%)	94 (9.4%)
Chronic respiratory diseases	47 (8.5%)	33 (7.4%)	80 (8.0%)
Mental illness (including depression)	54 (9.7%)	10 (2.2%)	64 (6.4%)
Depression	42 (7.6%)	8 (1.8%)	50 (5.0%)
Malignancies	20 (3.6%)	17 (3.8%)	37 (3.7%)
Comorbidity without mental illness	342 (61.5%)	246 (54.9%)	588 (58.6%)
Anti-depressive therapy [n (%)]	65 (11.7%)	10 (2.2%)	75 (7.5%)

^a^ Number (%) of patients with non-missing data unless stated otherwise. Abbreviations: SD = standard deviation, RA = rheumatoid arthritis, DAS = Disease Activity Score, ESR = erythrocyte sedimentation rate, RAID = Rheumatoid Arthritis Impact of Disease questionnaire, cDMARDs = conventional Disease-Modifying Anti-rheumatic Drugs, bDMARDs = biological Disease-Modifying Anti-rheumatic Drugs, NSAIDs = Nonsteroidal Anti-inflammatory Drugs

### Prevalence of depressive symptoms in RA

Overall, the median PHQ-9 score was 5.0 (range 0.0–27.0). Based on the categorized results from the PHQ-9, 55.1% (95% CI: 51.89–58.23) (537 / 975 patients) of patients reported at least mild depressive symptoms (Table 2) with the following distribution across the predefined categories: 44.9% (none), 34.2% (mild), 15.5% (moderate), 3.6% (moderately severe), and 1.8% (severe). The median BDI-II score was 8.0 (range 0.0–56.0). Based on the categorized results from the BDI-II, 27.7% (95% CI: 24.91–30.63) (269 / 971 patients) of the RA patients had at least mild depressive symptoms (Table 2) while 20.4% had minimal depressive symptoms, which was below the predefined cutpoint of interest in this study. Point prevalence rates for depressive symptoms based on the BDI-II were as follows: 51.9%, (none), 20.4% (minimal), 15.7% (mild), 7.8% (moderate), and 4.2% (severe).

**Table 2:** Point prevalence of depressive symptoms by depression questionnaire(s).

	Point Prevalence % (n / total)	95% CI
**≥ Mild depressive symptoms**		
PHQ-9 Positive (PHQ-9 ≥5)	55.1% (537 / 975)	51.89–58.23
BDI-II Positive (BDI-II ≥14)	27.7% (269 / 971)	24.91–30.63
BDI-II ≥14 or PHQ-9 ≥5	55.4% (556 / 1004)	52.24–58.48
**≥ Moderate depressive symptoms**		
PHQ-9 Positive (PHQ-9 ≥10)	20.9% (204 / 975)	18.41–23.61
BDI-II Positive (BDI-II ≥20)	12.0% (117 / 971)	10.07–14.26
BDI-II ≥20 or PHQ-9 ≥10	22.8% (229 / 1004)	20.25–25.53

^a^ Number (%) of patients with non-missing data

Considering all patients with a BDI-II ≥14 or a PHQ-9 ≥5, the point prevalence of at least mild depressive symptoms based on one or the other questionnaire was 55.4% (95% CI: 52.24–58.48) (556 / 1004 patients; see Table 2). Using more restrictive cut-points indicating at least moderate depressive symptoms, i.e. BDI-II ≥20 or PHQ-9 ≥10, the point prevalence was 22.8% (95% CI 20.25–25.53) (229 / 1004 patients) (Table 2).

Patients with at least mild depressive symptoms were less likely to have ACR/EULAR Boolean remission than patients with no depressive symptoms (16.3% vs 37.2%), and other chronic pain was more frequently reported by these patients than patients with no depressive symptoms (13.1% vs 4.7%).

### Characteristics related to depressive symptoms in RA

The unadjusted ORs and the 95% CIs for the risk of depressive symptoms stratified by covariates suggested that age (<60 years), BMI (≥25), not being in DAS28 remission (DAS28 (ESR) ≥2.6), a RAID total score >2, steroid use, NSAID use, opiate use and presence of chronic pain lead to a higher risk of having depressive symptoms. Gender, RA disease duration, family status, education, employment, cDMARDs, bDMARDs and the presence of comorbidity excluding mental illness or chronic pain were not related to depressive symptoms in the univariate analyses. ORs obtained by multiple logistic regression which adjusted for all other covariates in the model, confirmed that age (<60 years), a RAID total score >2 and presence of chronic pain lead to a statistically significant higher risk of having depressive symptoms. In this context, not having RAID PASS (i.e. RAID total score >2) seemed to be the most important risk factor (OR: 10.54, 95% CI: 6.87–16.16) followed by presence of chronic pain (OR: 3.25, 95% CI: 1.65–6.39) and age < 60 years (OR: 1.78, 95% CI: 1.13–2.80). This pattern of results was found for the ORs given for both at least mild depressive symptoms (BDI-II ≥14 or PHQ-9 ≥5) and at least moderate depressive symptoms (BDI-II ≥20 or PHQ-9 ≥10), whereas the results for RAID PASS showed considerably increased ORs in both the univariate and multiple analyses (Table 3). Furthermore, the univariate RAID single item analysis addressing distinct symptoms indicated that all items were related to the occurrence of depressive symptoms (Table 4). The step-down multiple regression procedure verified significance of RAID score, chronic pain and age, and showed that NSAID medication was marginally significant for at least moderately depressive symptoms (OR: 1.47 95% CI: 1.02–2.13).

**Table 3:** Odds ratios of at least mild or moderate depressive symptoms by patient and RA-related characteristics.

	≥ Mild Odds Ratio (95% CI)		≥ Moderate Odds Ratio (95% CI)	
Influence Variable	Unadjusted	Adjusted	Unadjusted	Adjusted
Age (1: <60, 0: ≥60)	1.50 (1.16–1.93)	1.78 (1.13–2.80)	1.76 (1.31–2.37)	2.16 (1.31–3.56)
Sex (0 = female, 1 = male)	0.78 (0.59 - 1.04)	0.93 (0.63–1.36)	0.88 (0.62–1.25)	0.96 (0.62–1.50)
Family status (family = 1, alone = 0)	1.00 (0.74–1.34)	1.07 (0.72–1.59)	0.99 (0.70–1.42)	1.20 (0.75–1.93)
BMI (kg/m2) (≥25 = 1, <25 = 0)	1.49 (1.15–1.93)	1.09 (0.77–1.55)	1.27 (0.93–1.74)	1.02 (0.68–1.53)
Education (>10 years = 1, ≤10 years = 0)	0.97 (0.75–1.27)	0.98 (0.70–1.38)	0.80 (0.59–1.10)	0.75 (0.51–1.10)
Employment (employed = 1, other = 0)	1.09 (0.84–1.41)	1.14 (0.73–1.79)	1.22 (0.90–1.64)	1.11 (0.68–1.82)
RA Duration (years)	1.00 (0.99–1.02)	1.00 (0.98–1.01)	0.99 (0.97–1.00)	0.99 (0.97–1.01)
DAS28 (ESR) (<2.6, ≥2.6)	2.13 (1.61–2.80)	1.27 (0.90–1.80)	1.89 (1.37–2.61)	1.34 (0.91–1.97)
RAID Score (≤2, >2)	11.34 (7.94–16.21)	10.54 (6.87–16.16)	19.23 (8.43–43.86)	13.61 (5.80–31.92)
cDMARDs (yes = 1, no = 0)	0.90 (0.65–1.25)	0.88 (0.57–1.37)	0.75 (0.52–1.08)	0.62 (0.39–1.00)
bDMARDs (yes = 1, no = 0)	1.20 (0.93–1.54)	0.99 (0.69–1.42)	1.07 (0.79–1.44)	0.83 (0.55–1.26)
Glucocorticoids medication (yes = 1, no = 0)	1.54 (1.19–1.98)	1.03 (0.74–1.44)	1.50 (1.11–2.03)	1.02 (0.70–1.49)
NSAID medication (yes = 1, no = 0)	1.76 (1.35–2.30)	1.24 (0.87–1.75)	1.74 (1.29–2.35)	1.42 (0.97–2.08)
Opiates medication (yes = 1, no = 0)	3.03 (1.49–6.18)	1.50 (0.64–3.48)	2.77 (1.51–5.06)	1.67 (0.79–3.55)
Comorbidity without mental illness or chronic pain (0 = no, 1 = yes)	0.79 (0.62–1.02)	1.03 (0.71–1.49)	0.81 (0.60–1.09)	1.18 (0.77–1.81)
Chronic pain (0 = no, 1 = yes)	3.07 (1.86–5.08)	3.25 (1.65–6.39)	2.55 (1.64–3.97)	2.90 (1.55–5.43)

^a^Reference category for ORs: age ≥60, female sex, DAS28 <2.6, RAID ≤2, no for each type of RA medication, and no for presence of a comorbidity without mental illness or chronic pain, no for chronic pain. Adjusted odds ratios adjusted in multiple logistic regression for other covariates in table. Abbreviations: RA = rheumatoid arthritis, DAS = Disease Activity Score, ESR = erythrocyte sedimentation rate, RAID = Rheumatoid Arthritis Impact of Disease questionnaire

**Table 4:** Risk of depressive symptoms (≥ Mild, ≥ Moderate) for RAID single item scores.

RAID single item scores^a^	Unadjusted Odds Ratio	95% CI	Unadjusted Odds Ratio	95% CI
	≥ Mild		≥ Moderate	
Pain	1.40	1.31–1.49	1.37	1.28–1.46
Functional disability assessment	1.45	1.36–1.55	1.42	1.32–1.52
Fatigue	1.74	1.62–1.87	1.64	1.52–1.77
Sleep	1.50	1.42–1.59	1.46	1.38–1.56
Physical well-being	1.63	1.52–1.75	1.57	1.45–1.69
Emotional well-being	2.31	2.08–2.56	1.95	1.78–2.14
Coping	1.81	1.67–1.96	1.67	1.55–1.81

^a^ Score range: 0–10. “Unadjusted” refers to the univariate relation of the independent variable to the dependent variable in logistic regression.

## Discussion

To the best of our knowledge, this is the first time the RAID questionnaire and its PASS criterion have been applied to a study addressing depressive symptoms in RA. Furthermore, this is the first time depression questionnaires that were previously validated specifically in RA patients have been used in a prevalence study.

In summary, our findings show that depressive symptoms measured with previously validated questionnaires are common in RA, with frequencies ranging from 12.0% (at least moderate, BDI-II) to 55.1% (at least mild, PHQ-9), depending on the respective category threshold and the questionnaire’s level of detail. In both severity categories of interest, the BDI-II as tool with a higher level of detail, returned lower frequencies of depressive symptoms compared to the PHQ-9. Moreover, the frequency of depressive symptoms on the PHQ-9 in the present study (20.9% at least moderate symptom severity) was lower than in a corresponding meta-analysis (38.8%) [11]. In our sample, patients lacking PASS according to the RAID questionnaire, patients less than 60 years of age, or patients with chronic pain were prone to depressive symptoms. The RAID PASS result clearly supports previous findings that highlight the relationship between pain, fatigue, quality of sleep, coping (all measured in the RAID), and depressive symptomology [26–33].

Applying the BDI-II or the PHQ-9 along with the RAID in clinical routine is likely to benefit patients and rheumatologists not only due to an improved detection of depressiveness but also by shared decision making with the help of structured patient-reported outcomes incorporated into the clinical evaluation. A point that still is frequently neglected in clinical routine. Outcomes such as the RAID or the PHQ-9 may explain why patients with RA, despite clinical remission, still might show impaired emotional or physical well-being.

Chronic pain, another explanation for considerably reduced well-being, also increased the risk of depressive symptoms in our sample. Previous research has shown that inflammation (with pain as a prominent component) might be triggering depression. Nevertheless, further proof is needed to determine whether chronic pain is a predictor of depression in RA, vice versa, or whether the relationship is bidirectional [34–38]. In our sample, moderate depressive symptoms occurred twice as frequently in patients below 60 years of age compared to patients aged 60 years and above. A previous meta-analysis found similar results [11]. Accordingly, depression seems to be more common in phases of personal life frequently characterized by founding a family, raising children, career development, establishing a work-life balance, and maintaining health while still working–all of that while facing the everyday burden of RA. Other commonly discussed covariates such as sex, drug class of anti-rheumatic medication, comorbidities other than chronic pain and mental illness, or DAS28 remission of RA could not be confirmed to be related to depressive symptoms.

For follow-up on borderline scores, the authors of the PHQ-9 suggest that not all patients showing mild depressive symptoms require taking action [14]. However, only 20.7% of our study participants with positive test results for moderate depressive symptoms in both questionnaires were previously diagnosed with depression or another mental disorder. Moreover, merely 11.7% of the patients classified as having depressive symptoms were receiving anti-depressive treatment (including psychotherapy) at the time of questioning. This is a clear call for proactive depression screening. Rheumatologists regularly see patients for routine check-ups and follow them up, and depression or emotional disturbances can impair RA treatment adherence [4, 5] [34, 39]. Thus, the rheumatologist’s role in screening for depressive disorders is crucial for both physical and mental well-being. Nevertheless, screening for depression only makes sense when coupled with follow-up routines for diagnostic workup and initiation of anti-depressive therapy [40, 41]. Thus, before the initiation of screening routines, rheumatologists should collaborate with mental-health professionals and/or therapy coordination services to establish efficient transfer of patients with depressive symptomology. The screening workflow should fit the clinical routine and address results indicating depressiveness at the very same patient visit. There should also be routines planned in case of a patient reporting suicidal ideations. This is especially pertinent for resident rheumatologists without instant access to the hospital environments.

Although we were able to recruit a patient sample from a wide variety of rheumatologic facilities in Germany, the reproducibility of our results and their practical application may be limited to the situation in Western Europe. Worldwide generalization of our results may not be possible due to differences in culture, health care system and limited availability of anti-rheumatic treatment. The low disease activity in our sample (e.g. median ESR: 14mm/h, median CRP: 0 mg/dl, median TJC: 0, median SJC: 0)–likely the result of adequate clinical care and sufficient access to effective bDMARDs–may accurately reflect treatment at German centres, but may also limit the generalizability of these results to large populations of patients with severe RA and patients with early or persistent RA. Furthermore, to ensure comparability with other studies, analysis of these data used standard PHQ-9 ≥5 and BDI-II ≥14 cut-offs to determine mild depressive symptomology. Validation of these instruments specifically in RA patients found mild depressive symptomology cut-offs to be PHQ-9 ≥6 and BDI-II ≥12 [17].

Overall, we think that these data are robust and therefore translate to other countries with good access to anti-rheumatic therapy. Especially as these data show that despite good control of inflammatory symptoms of RA, comorbid depression constitutes a significant burden for patients. Coupling depression screening routines with consultation by a mental health professional at the same visit may already suffice to lower unrecognized episodes of depression and improve clinical outcomes.

## Supporting information

S1 Table VADERA II—Raw data. (XLSX)
